# Clinical significance of the histological and molecular characteristics of ependymal tumors: a single institution case series from China

**DOI:** 10.1186/s12885-019-5877-9

**Published:** 2019-07-19

**Authors:** Shaoyan Xi, Ke Sai, Wanming Hu, Fang Wang, Yinsheng Chen, Jing Wang, Jing Zeng, Zhongping Chen

**Affiliations:** 1 Department of Pathology, Sun Yat-sen University Cancer Center, State Key Laboratory of Oncology in South China, Collaborative Innovation Center for Cancer Medicine, Guangzhou, 510060 China; 2 Department of Neurosurgery/Neuro-oncology, Sun Yat-sen University Cancer Center, State Key Laboratory of Oncology in South China, Collaborative Innovation Center for Cancer Medicine, 651 Dongfeng Rd. East, Guangzhou, 510060 China; 3 Department of Molecular Diagnostics, Sun Yat-sen University Cancer Center, Collaborative Innovation Center for Cancer Medicine, Guangzhou, 510060 China

**Keywords:** Ependymal tumor, Histological characteristics, RELA, Prognosis, H3K27me3

## Abstract

**Background:**

Ependymal tumors are pathologically defined intrinsic neoplasms originating in the intracranial compartments or the spinal cord that affect both children and adults. The recently integrated classification of ependymomas based on both histological and molecular characteristics is capable of subgrouping patients with various prognoses. However, the application of histological and molecular markers in Chinese patients with ependymomas has rarely been reported. We aimed to demonstrate the significance of histological characteristics, the v-relavian reticuloendotheliosis viral oncogene homolog A (RELA) fusions and other molecular features in ependymal tumors.

**Methods:**

We reviewed the histological characteristics of ependymal tumors using conventional pathological slides and investigate the RELA fusions and Cylclin D1 (CCND1) amplification by Fluorescence in situ hybridization (FISH) and trimethylation of histone 3 lysine 27 (H3K27me3) expression by immunohistochemistry (IHC) methods. SPSS software was used to analyze the data.

**Results:**

We demonstrated that hypercellularity, atypia, microvascular proliferation, necrosis, mitosis, and an elevated Ki-67 index, were tightly associated with an advanced tumor grade. Tumor location, necrosis, mitosis and the Ki-67 index were related to the survival of the ependymomas, but Ki67 was the only independent prognostic factor. Additionally, RELA fusions, mostly presented in pediatric grade III intracranial ependymomas, indicated decreased survival times of patients, and closely related to the patients’ age, tumor grade, cellularity, cellular atypia, necrosis and Ki67 index in the intracranial ependymal tumors, whereas reduction of H3K27me3 predicted the worse prognosis in ependymal tumors.

**Conclusions:**

Histological and molecular features facilitate tumor grading and prognostic predictions for ependymal tumors in Chinese patients.

## Background

Ependymomas are rare tumors originating from the ependymal epithelium throughout the neuroaxis and account for 3–10% of all neuroepithelial tumors. Although ependymomas can occur in patients at any age, children and young adults are prevalently affected. Ependymal tumors include sub-ependymomas, myxopapillary ependymomas and ependymomas. The prognoses of ependymal tumors are not identical and may depend on the extent of surgical resection, adjuvant therapy, and the molecular characteristics [1, 2]. Classical ependymomas and anaplastic ependymomas correspond to the WHO grades II and III, whereas variants such as myxopapillary ependymomas or sub-ependymomas correspond to the WHO grade I tumors [3]. However, the criteria for grading ependymomas are not well established [4–6]. Few studies have investigated the prognostic significance of the WHO grade or individual pathological features across large trial cohorts. For the reasons above, the management of ependymomas is controversial due to uncertain biological behavior.

Genetic studies have shown that molecular alterations are very common in ependymomas, which display a broad range of cytogenetic aberrations [7, 8]. Ependymal tumors are divided into three subgroups (supratentorial, posterior fossa and spinal cord) according to the tumor location and nine molecular subgroups. One of the nine molecular alterations driving major prognostic implications is v-relavian reticuloendotheliosis viral oncogene homolog A (RELA)fusions [9]. RELA fusions often refer to as C11ORF95-RELA fusions. The RELA gene is located in 11q, and RELA fusions activate the Nuclear factor kappa B (NF-κB) cellular pathway, which is a central mediator of the cellular inflammatory response [10, 11]. The NF-κB signaling is observed in most human cancers. RELA fusions have been shown to activate the NF-κB target gene, upregulate L1 cell adhesion molecule (L1CAM) [2] and thereby have a profound impact on the expression of several other genes that regulate focal adhesion [12, 13]. In a study by Mathew Parker et al. [9], the sequencing data of ependymomas cases showed amplification of CCND1in 18/89 cases. Additionally, since CCND1 is a direct transcriptional target of NF-κB signaling, it has the potential to contribute to the onset and/or progression of oncogenesis [14]. However, the significance of CCND1 amplification in ependymomas is not yet well studied.

Trimethylation of histone 3 lysine 27 (H3K27me3) is driven by aberrant DNA methylation and was used as a surrogate marker to distinguish between ependymomas posterior fossa group A (EP-PFA) and group B (EP-PFB) in a study of Panwalka [15]. H3K27me3 staining was found to be globally reduced in EP-PFA tumors, and immunohistochemistry (IHC) showed a sensitivity and specificity of 99% and 100%, respectively, in segregating EP-PFA from EP-PFB tumors. H3K27me3 could be used to detect the DNA methylation of ependymomas in the routine practice as IHC is more convenience to perform than CpG island sequencing. H3K27me3 was also studied in high-grade gliomas and was found to have both diagnostic and biological significance. Decreased H3K27me3 was specific to a H3F3A K27 M mutant in Glioblastoma multiform (GBMs) while all other tumor subtypes, including H3F3A wild-type GBMs, showed strong H3K27me3 staining [16].

The combination of histological and molecular characteristics has pressing needs for deserving further research to improve the diagnosis and tailor treatment of ependymomas. In the current study, we investigated the histology and molecular markers of 69 ependymomas cases. We then performed Fluorescence in situ hybridization (FISH) to detect RELA gene fusions and explore the relationship between this variable and clinicopathological parameters. CCND1 was also examined by FISH to explore its prognostic value for ependymal tumor patients.H3K27me3 expression in ependymal tumors was also examined by using IHC and explored the prognostic value.

## Methods

### Patients

The data of patients received surgical treatment from January 2000 to December 2017 in our center and the surgical specimen diagnosed histologically as ependymal tumors were collected. Patients who receive therapy before surgery or died of diseases unrelated to ependymomas were excluded. A cohort of 69 cases was retrieved and this current study was approved by the ethics committee at our center. Written informed consent was obtained from all patients and/or their family members.

All cases were reviewed by two pathologists who were specialized in neuropathology. The histopathological characteristics evaluated comprised of cellularity, cellular atypia, necrosis, vascular proliferation, and mitosis.

### IHC staining

IHC staining was performed following the standard procedures as briefly mentioned in one of our previous study [17]. Briefly, tissue slides were dewaxed and dehydrated according to our standard protocol. Antigen retrieval was performed by boiling the slides in antigen retrieval buffer for 15 min. A primary antibody targeting Ki67 (DAKO, Denmark, MIB-1, Ready to use) and H3K27me3 (Abcam, Cambridge, UK, ab6002, 1:100) were added to the section and incubated at 4 °C overnight. A secondary antibody (DAKO, Denmark, ready to use) was added and incubated for 1 h after rinsing with PBS. DAB (DAKO, Denmark), was used for staining. H3K27me3 and Ki67 were positive for nucleus.

### FISH analysis

FISH analysis was performed to detect the CCND1 gene copy number and RELA rearrangement status. The FISH assay included the CCND1(11q13)/CSP11 probe and RELA (11q13) break apart rearrangement probe (LBP Medicine Science and Technology Co., Ltd., Guangzhou, China). Formalin-fixed, paraffin-embedded, 4-μm-thick sections were used for FISH detection. The detailed FISH staining procedures was performed as we described previously. Hybridization signals for each probe were assessed under an Olympus BX51 TRF microscope (Olympus, Tokyo, Japan) equipped with a triple-pass filter (4′,6′-diamidino-2-phenylindole, DAPI/Green/Orange; Abbott Molecular Inc., Des Plaines, IL, USA). The scoring was conducted in no less than 50 non-overlapping nuclei per core in tumor regions. Tumors were considered to be present for rearrangement if more than 15% of the nuclei demonstrated separate red and green signals. The average copy number of CCND1 and the chromosome11 centromere signal were determined, and a CCND1/CSP11 ratio was calculated for each case. Tumors with≥4 CCND1 signals per cell or a ratio ≥ 2.0 were classified as CCND1 amplification.

### Statistical analysis

The Statistical Product and Service Solutions (SPSS) statistical package for Window version 25 (SPSS, Chicago, IL, USA) was used for data analyses. The survival curves of ependymomas patients were derived with the Kaplan-Meier method. Multivariate analysis was performed by Cox regression. The correlation between clinical features of ependymomas patients and the tumor grade was analyzed using Pearson’s χ^2^ test and ROC curves. The ROC curves were also used to identify the cutoff values for mitosis and the Ki67 index. A *P* value < 0.05 was considered to show statistical significance.

## Results

### Case cohort

A total of 69 patients were enrolled in the current study. Among them, they were 35 males and 34 females. The mean age at diagnosis was 25 years, and 26 patients were under 18 years old. Twenty-eight ependymomas originated in the supratentorial areas, 18 in the posterior fossa and 23 in the spinal cord. Thirty-eight, 29 and 2 cases were diagnosed as grade III, II and I, respectively. Total resection was achieved in 52 out of 69 cases. Forty-eight patients were treated with surgery only, 8 had plus radiotherapy, while 13 had surgery, followed by adjuvant radiotherapy and chemotherapy. The patients were followed up for 1 to 200 months. Eleven patients died from their tumors at the time of final follow-up.

### Histology-tumor grading

**Fig. 1:**
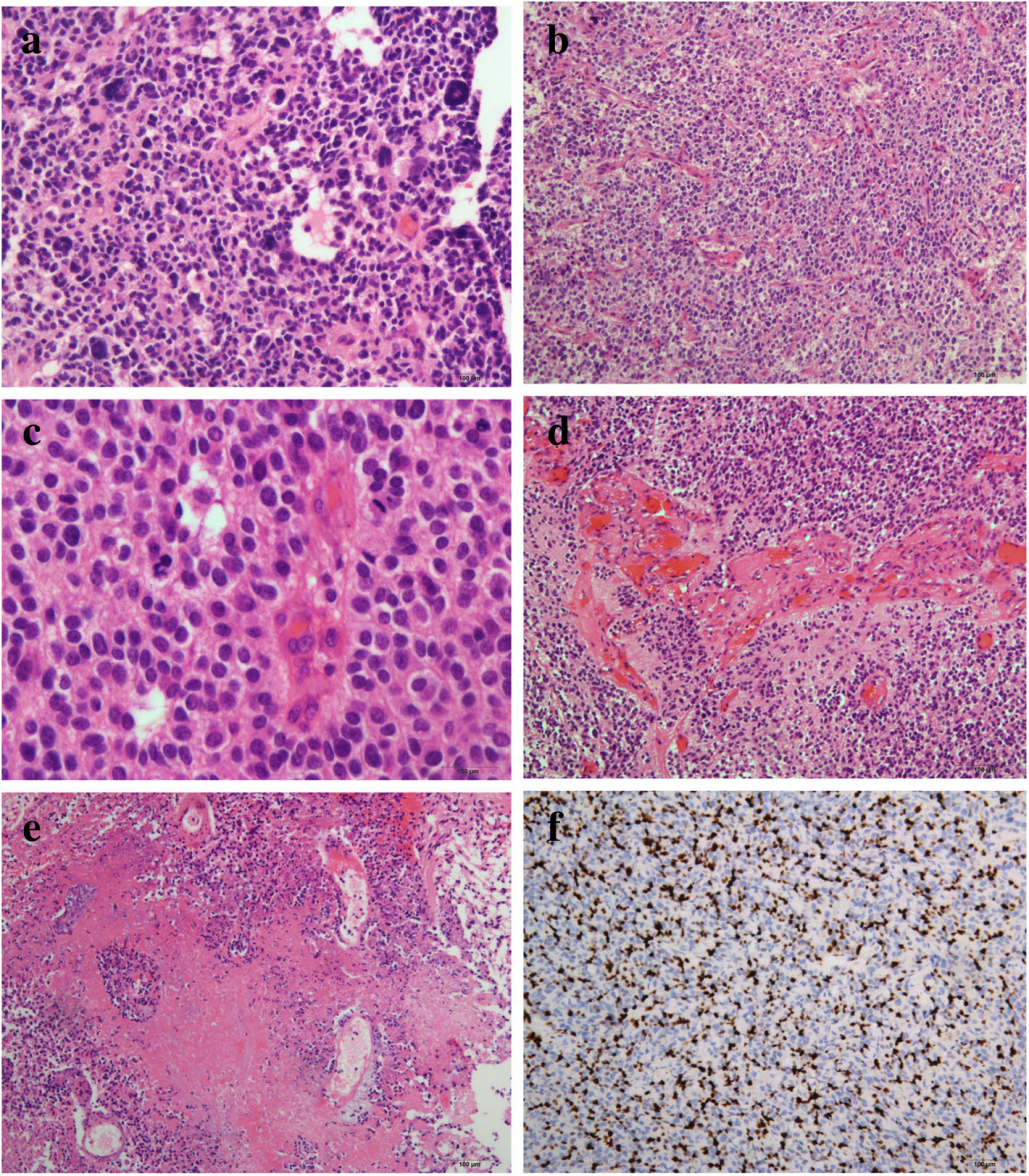
(**a**) The figure shows pleomorphic tumor cells with nuclear atypia and multinucleated giant cells of the ependymal tumor; the bizarre nuclei were eye-catching; (**b**) The figure shows the high cellularity of the tumor, with crowding of the tumor cells; (**c**) The figure shows brisk mitosis in the tumors, with mitotic events counted in 2 mm^2^ high-power fields; (**d**) The figure shows microvascular proliferation, which often presents with a glomeruloid appearance; (**e**) The figure shows tumor necrosis, but palisading necrosis is easily seen; and (**f**) The figure shows the high Ki67 index in the ependymal tumors

**Fig. 2:**
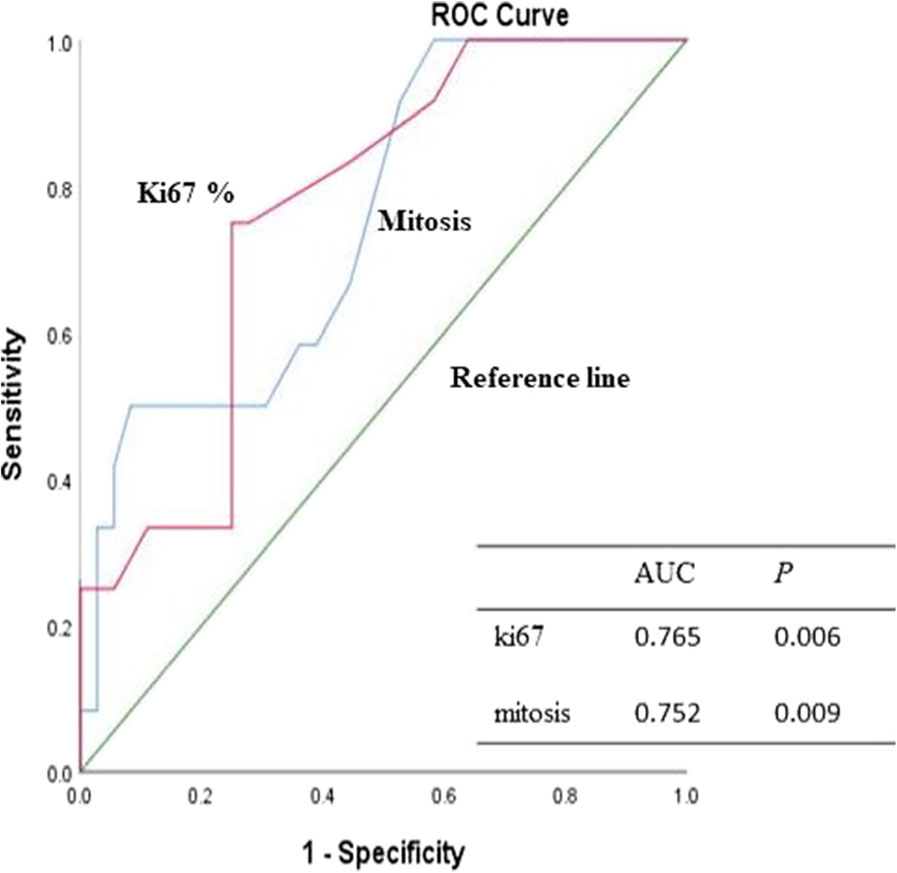
ROC curve was used to identify the cutoff points for Ki67 and mitosis, with AUCs (areas under the curve) of 0.765 and 0.752, respectively. The *P* values were all < 0.05. The cutoff points detected by the ROC curves for Ki67 and mitosis were 6% and 5/2mm^2^, respectively

All cases were reviewed by two pathologists who were specialized in neuropathology.. As shown in Table 1 and Fig. 1, cellularity, cellular atypia, vascular proliferation, necrosis, mitosis and the Ki67 index were associated with the tumor grade. Correlative analyse showed that RELA fusions were significantly related to the higher tumor grade because the most of RELA fusions events (16/17) occurred in grade III ependymal tumors. Additionally, the cutoff points for mitosis and Ki67 were 5/2mm^2^and 6%, respectively, which was used to identify grade III tumors (Fig. 2).

**Table 1:** The relationship between the pathological parameters and higher tumor grading

		Grade III cases^a^	*P* value
Cellularity	low	6/15	0.037
	moderate	10/16	
	high	19/22	
Atypia	mild	8/18	0.049
	moderate	13/20	
	severe	13/15	
Necrosis	present	22/24	<0.001
	absent	12/29	
Mitosis	≥5/2mm^2^	18/21	0.016
	<5/2mm^2^	16/32	
Vascular proliferation	Present	19/21	0.002
	Absent	15/32	
Ki67 index	≥6%	19/20	<0.001
	< 6%	14/38	
RELA fusions	Positive	17/19	0.001
	Negative	20/48	

^a^: Some cases were lost due to inadequate tissue for testing or other reasons

### Prognostic value of clinicopathological parameters in ependymal tumors

**Fig. 3:**
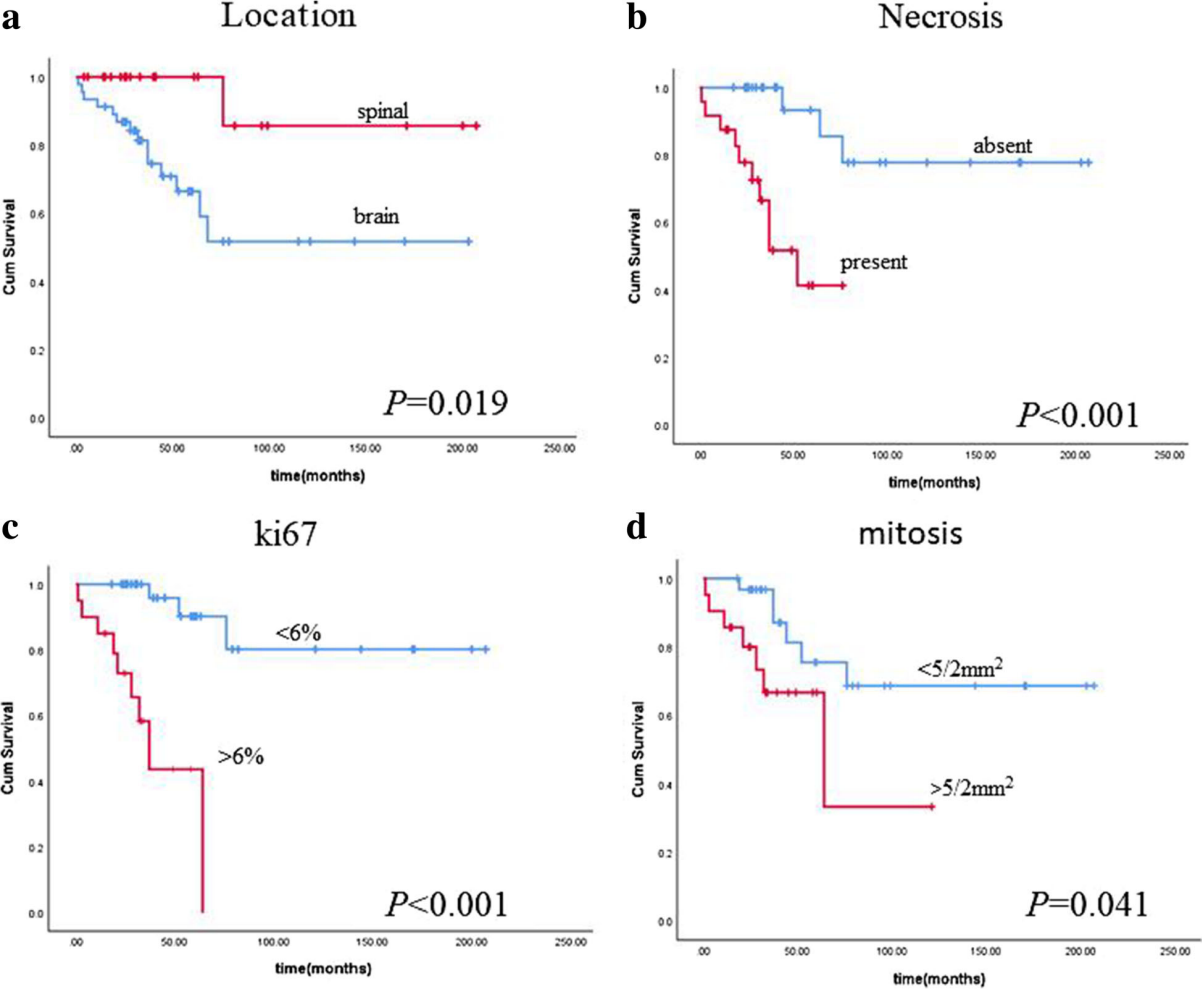
Kaplan-Meier analysis showed that tumor location, necrosis, high Ki67 and high Ki67 mitotic activity were related to shorter survival times in ependymomas patients (**a**:124.06 ± 17.31 months vs 188.29 ± 17.31 months, *P* = 0.019, **b**:174.82 ± 16.47 months vs 48.28 ± 6.07 months, *P* < 0.001; **c**:179.05 ± 15.76 months vs 40.92 ± 6.35 months, *P* < 0.001; **d**: 157.03 ± 17.36 vs 67.58 ± 15.88 months, *P* = 0.041;respectively)

Univariable analyses were performed to investigate the prognostic factors related to survival. As shown in Fig. 3, the Kaplan-Meier analysis showed that tumor location (intracranial ependymomas or spinal ependymomas), necrosis, high Ki67and high mitotic activity were related to shorter survival times in ependymomas patients (124.06 ± 17.31 months vs 188.29 ± 17.31 months, *P* = 0.019; 174.82 ± 16.47 months vs 48.28 ± 6.07 months, *P* < 0.001;179.05 ± 15.76 monthsvs40.92 ± 6.35 months, *P* < 0.001; and 157.03 ± 17.36 vs 67.58 ± 15.88 months, *P* = 0.041;respectively). Cox regression analysis showed the Ki67 was the only independent biomarker for the prognosis in ependymal tumors (Table 2).

**Table 2:** Cox regression model to analyze the overall survival in ependymomas

Clinicopathologic features	Multivariate analysis		
	Risk ratio	95% CI	*P*
Tumor location	0.898	0.279–2.891	0.854
necrosis	166,316.130	0.000–3.995E+ 142	0.941
mitosis	0.515	0.128–2.071	0.350
Ki67 index	8.538	1.158–62.958	0.035*

CI, confidence interval, *P < 0.005 represents statistically significant

### RELA fusions related to clinicopathological parameters and predicted a worse prognosis

**Fig. 4:**
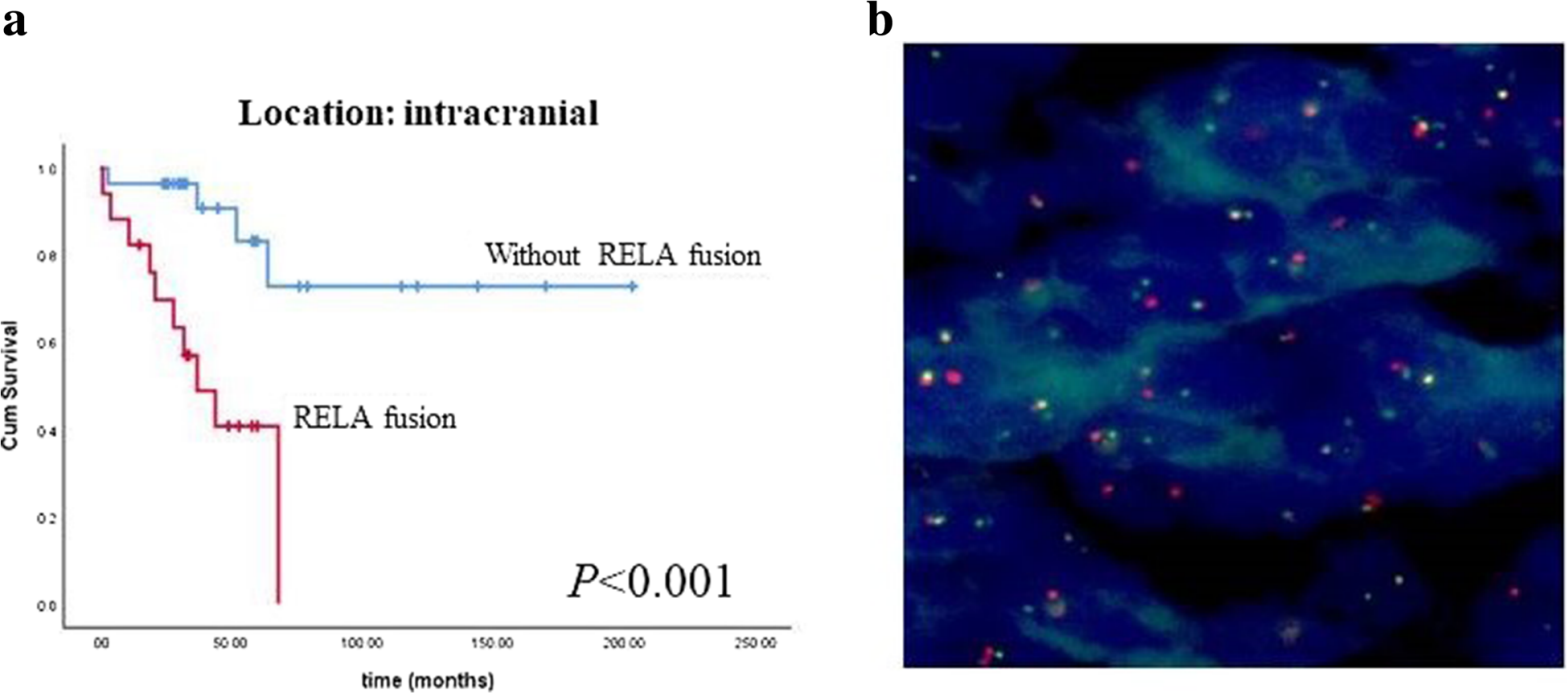
Kaplan-Meier analysis showed that the RELA gene fusion group had shorter survival for intracranial ependymal tumors (*P* <0.001) (**a**); RELA fusions were detected using the RELA break apart probe (**b**)

RELA fusions were mostly present in intracranial ependymomas (17/19) and were significantly correlated with the age, tumor grade, cellularity, cellular atypia, necrosis and the Ki67 index in the supratentorial ependymal tumors (Table 3). RELA fusions were also significantly related to a shorter survival time in intracranial ependymomas (*P* < 0.001) (Fig. 4).

**Table 3:** Clinicopathological parameters correlated to RELA fusions in intracranial ependymal tumors

	RELA fusions present	*P* value
Age		0.002
≤ 18 years	13/21	
> 18 years	4/24	
Gender		
Male	11/25	0.336
Female	6/20	
WHO grade		
Grade II	1/15	0.008
Grade III	16/30	
Cellularity		
low	1/5	0.047
moderate	2/12	
high	11/19	
Cellular atypia		
mild	1/9	0.029
moderate	4/13	
severe	9/14	
Vascular proliferation		
absent	3/19	0.003
present	11/17	
Mitosis		
≤ 5/2mm^2^	4/19	0.020
> 5/2mm^2^	10/17	
Necrosis		
absent	2/15	0.008
present	12/21	
Ki67 index		
≤ 6%	2/23	<0.001
> 6%	12/16	
CCND1 amplification		
present	5/14	0.800
absent	8/20	

*: Some cases were lost due to inadequate tissue for testing or other reasons

### CCND1 amplifications in ependymal tumors

**Fig. 5:**
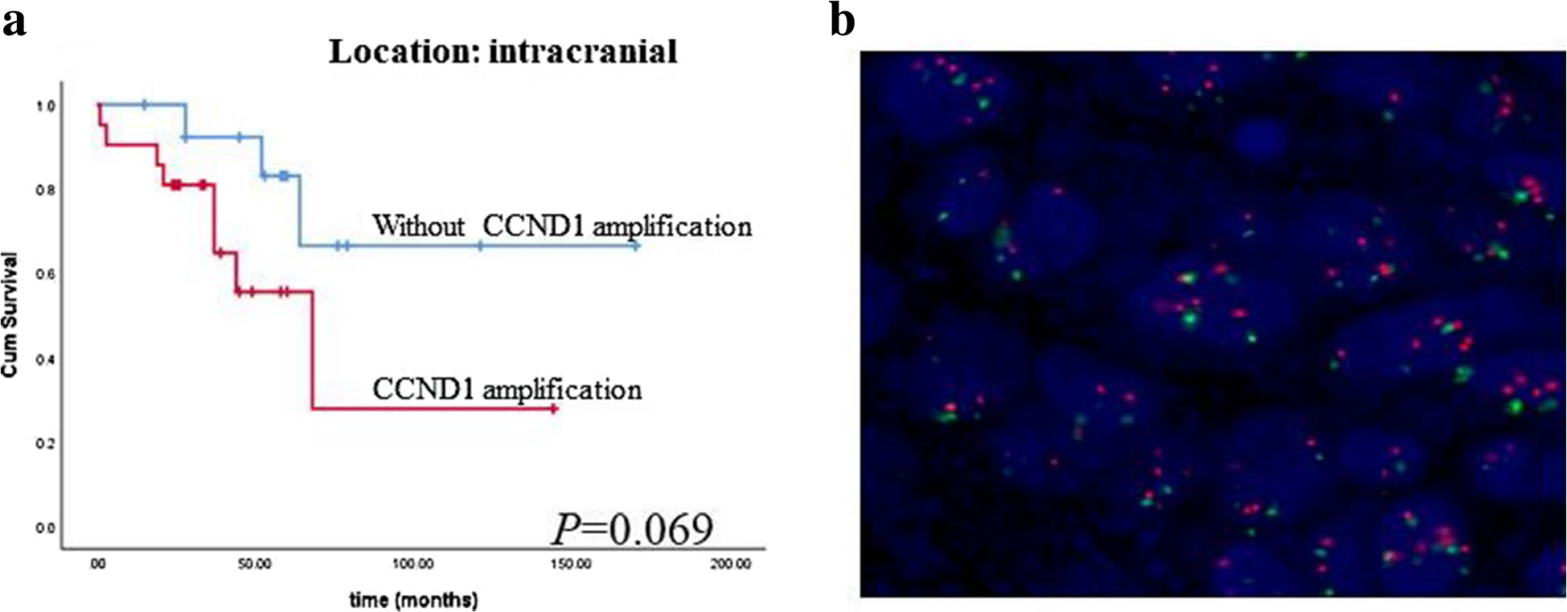
Fig. 5 (**a**) Kaplan- Meier analysis showed that ependymal tumors with CCND1 amplifications had shorter survival in the intracranial ependymal tumors (P =0.069) Figure 5b showed that the CCND1 amplifications

There were 22 (22/54) cases showing CCND1 amplification by FISH test. We also found that CCND1 amplification might predict a shorter survival (159.44 ± 20.12 months vs 90.74 ± 17.03 months, *P* = 0.125). In further analyses, we found that the prognostic value of CCND1 amplifications in intracranial ependymomas was more distinct, although no statistical significance (Fig. 5).

### The prognostic value of H3K27me3 loss in ependymomas

**Fig. 6:**
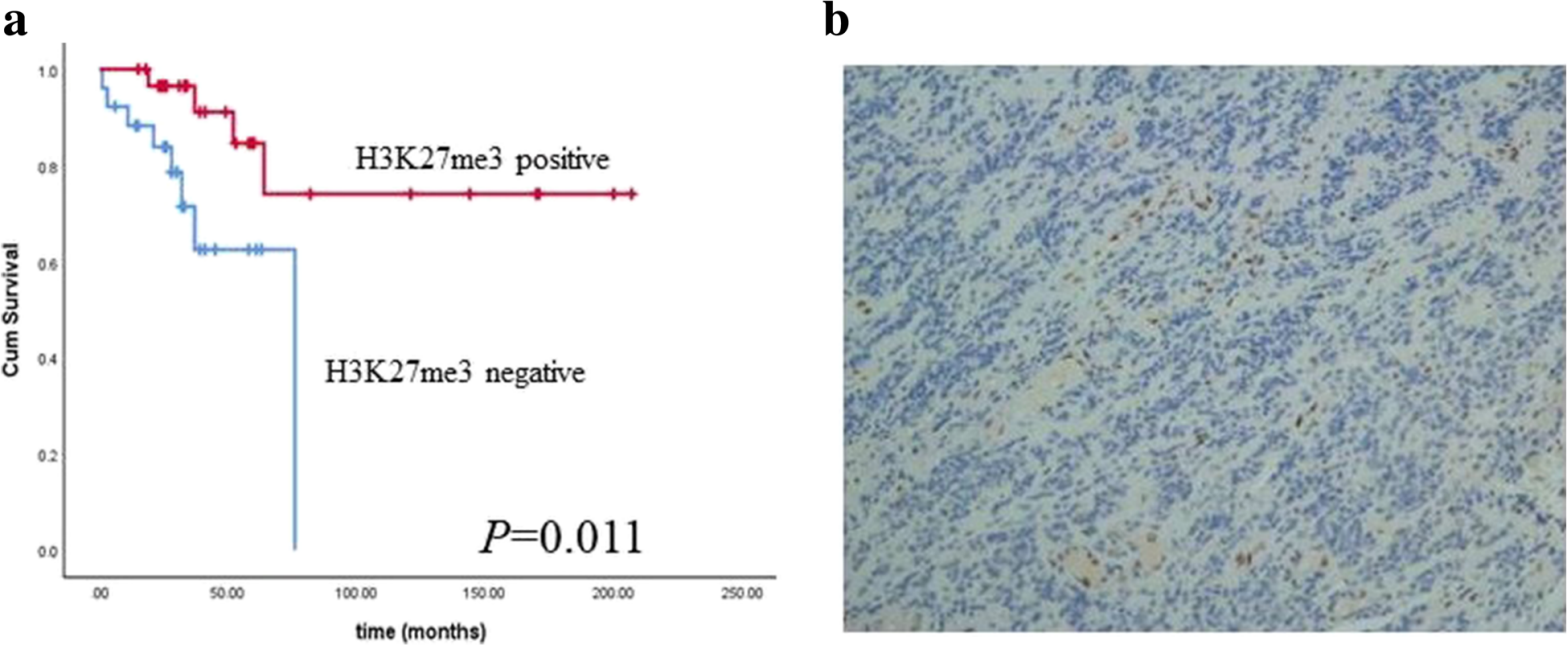
Kaplan-Meier analysis showed that ependymal tumors with loss of H3K27me3 had shorter survival (56.12 ± 6.67 months vs 166.17 ± 18.88 months, *P* = 0.011) (**a**). Figure 5**b** showed that the loss of H3K27me3 expressions, with the endothelia serving as an inner positive control

We performed IHC to evaluate H3K27me3 expression in ependymal tumors. H3K27me3 was absent in tumor cells but was preserved in endothelial cells and infiltrating lymphocytes in 25/58 cases. As shown in Fig. 6, the Kaplan-Meier analysis showed that a loss of H3K27me3 expression predicted a worsening prognosis (56.12 ± 6.67 months vs166.17 ± 18.88 months, *P* = 0.011, Fig. 6).

## Discussion

The WHO system for grading of primary brain tumors including ependymomas can classify the low and high grade tumors as grades II or III, having survival time of ≥5 years or < 5 years, respectively [18, 19]. The histological grade of ependymomas has been reported to be an independent prognostic indicator for event-free survival [3]. However, pathological parameters used for grading such as cell density, nuclear pleomorphism, mitotic activity and Ki67 index were prone to considerable interobserver variability, and not meet a consensus [20]. In our study, we proved that cellular atypia, vascular proliferation, necrosis and the Ki67 index were associated with tumor grade. As our experience, these pathological parameters reflected the activity of the tumor, indicating the tumor growth rate. Among the parameters, mitosis count and the Ki67 index were more objective and reproducible [21]. Mitotic activity and Ki67 was commonly used for the assessment of cell proliferation rate in virtually any field of tumor pathology [20]. Stefan Wolfsberger et al. used a Ki67 cutoff value of 20.5% to distinguish between grade II and grade III tumors [20],. However, in the experiences of usual pathological work, 20.5% is considered as a too high value for a cutoff point to differentiate between the grade II and III tumors. In the present study, we generated ROC curves for identification of cutoff points for the mitosis count and Ki67 index, found as were 5 per 2 mm^2^ and 6%, respectively. The cutoff points that we made were more close to our usual work. Additionally, location, necrosis, mitosis and Ki67 index were related to prognosis, but only Ki67 index was found to be an independent biomarker for survival. We demonstrated that the clinicopathological parameters were also significance for the prognostic prediction.

Parker et al. extensively demonstrated that C11orf95-RELA fusions resulted from chromothripsis of chromosome 11q based on whole-genome sequencing data and direct transcriptional targeting of NF-κB signaling [9]. The NF-κB family of transcriptional regulators is central mediators of the cellular inflammatory response.[10] Therefore, the predictive value of RELA fusions for outcome attracted the attentions. In the study of Marco Gessi [22], the authors failed to demonstrate the prognostic value of RELA fusions, while Figarella-Branger et al. reported RELA fusions predicted a better survival rate [23]. The finding of this present study showed that RELA fusions were related to a shorter survival time in intracranial tumors. The rate of RELA fusions accounting for ST-EPN was range 40 to 70% [9, 22]. In our series, most of the RELA fusions (17/19) occurred in intracranial ependymomas, 37.8%(17/45) of intracranial had RELA fusions, and they were related to a younger age, aggressive histopathological features. We firstly reported that the RELA fusions were related to cellularity, cellular atypia and necrosis. Malgulwar Prit Benny et al. hypothesized that RELA fusions leaded to the increased vascularity and clear cells due to up-regulation of VEGF secondary by increased NF-κB signaling [24]. Figarella-Branger D showed that pathological NF-kB activation by this mechanism characterized human Supratentorial clear cell ependymomas with branching capillaries, which therefore represent a subset of ST-EPN-RELA. The aggressive pathological features related to RELA fusions may be induced by the aberrant NF-kB signaling, and the mechanism required further exploration.

CCND1 amplification has been reported in many cancers, including breast cancer, esophageal cancer, laryngeal, and lung cancers [25–30]. Whole gene sequencing of ependymomas identified CCND1 amplifications in some cases [9]. However, the clinical significance of CCND1 amplification is not well established. We detected CCND1 amplification in our clinical samples and found that CCND1 amplification was related to a shorter survival time especially in the intracranial cases. The survival trend indicated that CCND1 might cause malignancy in ependymomas.

H3K27me3 is a critical mark for stem cell maintenance and is mediated by EZH2, which is a member of the polycomb group (PcG) family [15]. H3K27 trimethylation plays a role in the repression of lineage regulatory genes during pluripotency in embryonic stem cells [31]. Furthermore, H3K27me3 is known to affect pathogenesis in H3F3A K27 M m DNA methylation [32]. Global loss of H3K27 trimethylation may contribute to pediatric GBM by affecting differentiation pathways [16]. H3K27me3 can also be used as a surrogate for DNA methylation; a reduction of H3K27me3 corresponds to EPN-PFA and suggests that the patients need post-surgery therapy and have a worse survival status [15]. In our study, we found that H3K27me3 could also be used as a prognostic marker for ependymal tumors. Therefore, our study suggests that the reduction of H3K27me3 may also participate in the epigenetic process in ependymal tumors.

## Conclusions

In this study of our single institutional series, the prognostic factors of ependymal tumors included conventional pathological features such as Ki67 index and newly discovered molecular markers including RELA gene fusion and H3K27me3. Ependymal tumor grading could be classified by pathological characteristics, and the combination of traditional pathological parameters and molecular approaches could provide a better evaluation of ependymal tumors.

## Data Availability

The authenticity of this article has been validated by uploading the key raw data onto the Research Data Deposit public platform (www.researchdata.org.cn), with the approval RDD number as RDDB2019000580.

## Abbreviations

CCND1: Cylclin D1
EP-PFA: Ependymomas posterior fossa group A
EP-PFB: Ependymomas posterior fossa group B
EZH2: Enhancer of zeste homolog 2
FISH: Fluorescence in situ hybridization
GBM: Glioblastoma multiform
H3K27me3: Histone H3 lysine 27 trimethylation
IHC: Immunohistochemical
L1CAM: L1 cell adhesion molecule
NF-κB: Nuclear factor kappa B
RELA: v-relavian reticuloendotheliosis viral oncogene homolog A
ST-EPN: Supratentorial ependymomas
WHO: World Health Organization
